# ROS and NO Phytomelatonin-Induced Signaling Mechanisms under Metal Toxicity in Plants: A Review

**DOI:** 10.3390/antiox10050775

**Published:** 2021-05-13

**Authors:** Miriam Pardo-Hernández, María López-Delacalle, José Manuel Martí-Guillen, Sara E. Martínez-Lorente, Rosa M. Rivero

**Affiliations:** Center of Edaphology and Applied Biology of Segura River—Spanish National Research Council (CEBAS-CSIC), Department of Plant Nutrition, Campus Universitario Espinardo, Ed. 25, 30100 Espinardo, Murcia, Spain; mpardo@cebas.csic.es (M.P.-H.); mlopez@cebas.csic.es (M.L.-D.); martiguillen18@gmail.com (J.M.M.-G.); sesperanza.martinez@um.es (S.E.M.-L.)

**Keywords:** metal toxicity, phytomelatonin, heavy metals, ROS, NO

## Abstract

Metal toxicity in soils, along with water runoff, are increasing environmental problems that affect agriculture directly and, in turn, human health. In light of finding a suitable and urgent solution, research on plant treatments with specific compounds that can help mitigate these effects has increased, and thus the exogenous application of melatonin (MET) and its role in alleviating the negative effects of metal toxicity in plants, have become more important in the last few years. MET is an important plant-related response molecule involved in growth, development, and reproduction, and in the induction of different stress-related key factors in plants. It has been shown that MET plays a protective role against the toxic effects induced by different metals (Pb, Cd, Cu, Zn, B, Al, V, Ni, La, As, and Cr) by regulating both the enzymatic and non-enzymatic antioxidant plant defense systems. In addition, MET interacts with many other signaling molecules, such as reactive oxygen species (ROS) and nitric oxide (NO) and participates in a wide variety of physiological reactions. Furthermore, MET treatment enhances osmoregulation and photosynthetic efficiency, and increases the concentration of other important antioxidants such as phenolic compounds, flavonoids, polyamines (PAs), and carotenoid compounds. Some recent studies have shown that MET appeared to be involved in the regulation of metal transport in plants, and lastly, various studies have confirmed that MET significantly upregulated stress tolerance-related genes. Despite all the knowledge acquired over the years, there is still more to know about how MET is involved in the metal toxicity tolerance of plants.

## 1. Introduction

Environmental degeneration, which is mainly caused by rapid industrial expansion (transport, mining and energy industries, and intensive agriculture) has become a major threat to living beings. Heavy metals are increasingly polluting the water and soil of industrialized areas [1], and although plants need trace amounts of metals, such as iron (Fe), copper (Cu), and zinc (Zn) to live, large amounts of them can induce severe stress on the plant, which directly affects plant productivity and, in turn, human health. The ratio of metal removal from soil by plants changes widely and is related to plant species, plant growth rate, and the concentration of heavy metals in the irrigation water or soil [2,3]. Currently, the most recent research interests are on finding plants known as heavy metal-hyperaccumulator plants, i.e., plants which have an extreme ability to accumulate one or more heavy metals [4,5,6], to provide an immediate solution for the recovery of contaminated soils.

In recent years, the exogenous application of melatonin (MET) has been shown as a possible solution to the cultivation of plants in contaminated soils and waters. MET is an antioxidant derivative of tryptophan that is mainly synthesized in mitochondria, chloroplasts, and to a lesser extent, in the cytosol [7,8] and its content in plants differs depending on cultivars, species, growth and developmental periods, tissue types, and even in repetitions from a single experiment [9,10]. Especially in plants, MET is maintained at a relatively constant level under normal conditions, just as with reactive oxygen species (ROS), and it is therefore believed that MET may mainly act as a regulator of ROS levels. Nevertheless, MET and ROS synthesis and accumulation can be greatly and rapidly activated and upregulated in response to stress conditions [11,12]. Several studies have reported that MET could be considered a growth regulator, as it plays a role in specific physiological processes in plants [13,14,15]. In addition, some results have demonstrated that MET could be useful in phytoremediation processes [2]. Previous studies have shown that an exogenous treatment with MET improved plant tolerance to abiotic stress such as drought, heat, and cold in plants [16]. However, the most important function that was recently described for MET in living organisms was related to its role in non-receptor-mediated enzymatic activities, such as those related to ROS and reactive nitrogen species (RNS) scavenging, which increased and improved the cell’s antioxidant capacity [9]. MET regulates the activity of different antioxidant enzymes and stress tolerance-related genes, and it is involved in the regulation of the downstream signaling transduction pathways in plants exposed to abiotic or biotic stress [17,18]. Also, MET improves the tolerance capacity of different plants cost-effectively and feasibly against environmental contamination, as it is considered an environmentally-friendly molecule. In this context, there is no evidence of serious MET toxicity in any plant, animal, or humans [19], although it has been shown that under special circumstances, high levels of MET may induce some growth inhibition [20].

Consequently, in the last few years, the study of exogenous MET application to plants to counteract metal toxicity, has increased. The first report in this area was published by Tan et al. [21], which correlated MET and N1-acetyl-N2-formyl-5-methoxykynuramine (AMFK, a melatonin derivative) contents with the tolerance capacity of water hyacinth plants against toxic pollutants [21]. The same research group demonstrated that the tolerance capacity of pea (*Pisum sativum*) plants significantly improved after supplementation with MET under Cu stress [19]. In general, it is believed that exogenous/endogenous MET improves metal toxicity tolerance capacities of plants by restricting metal mobility in the rhizosphere and aerial parts, along with boosting the activities of other key stress-related processes, such as vacuolar transporters, phytochelatins, and glutathione (GSH). These molecules are known to be further involved in the sequestration and detoxification of metals in plants, allowing for greater plant growth [22]. As a consequence, these results may offer an alternate use of MET in plants, as well as the improvement of human food safety.

MET functions have been comprehensively and deeply searched for in different plants under metal toxicity conditions. In this review, we summarize the most recent research conducted on metal toxicity in plants, and how MET treatments may improve metal stress tolerance in plants.

## 2. Role of MET in Plants Subjected to Metal Toxicity

The conditions under which plants usually grow are constantly changing, and plants are often subjected to various stress conditions. Therefore, improving plant stress resistance is not only critical for ensuring agronomic productivity, but also for environmental sustainability, as it has been demonstrated that crops with poor stress resistance consume more water and fertilizers [23]. Metal contamination is an important environmental problem, especially in areas with high anthropogenic pressure. Heavy metal accumulation directly influences crop growth due to phytotoxicity, agriculture due to the adverse effects of these metals on food safety and marketability, and the environmental health of soil organisms. As plants are an essential part of the ecosystem, and as they have a direct influence on the geological and biological redistribution of heavy metals that pollute the soil, water and air [24], knowledge on the effects of metal toxicity in plants and their response in such environments, is crucial for the development of improved agricultural traits and food production under these circumstances.

## Effects of Metal Toxicity in Plants

In general, high levels of metals (Pb, Cd, Cu, Zn, B, Al, V, Ni, La, As, and Cr) produce stunted growth and abnormal morphology, perturbations in water relations, ion metabolism, and mineral uptake, a decrease in photosynthetic rate, stomatal conductance, chlorophyll a and b biosynthesis, and an increase in chlorophyll degradation. These toxicities also induce a common oxidative burst in plants which causes an imbalance in the production and scavenging of ROS, which reduces photosynthesis and induces stomatal closure, alters the activities of many enzymes and lastly, produces cell damage and death. All of these effects have been shown to appear at high concentrations of Pb [25], Cd [26], Cu [27,28], Zn [29], B [30,31], Al [32,33,34], V [35], Ni [36,37,38], As [39,40,41], and Cr [42] in the growth media of the plants.

More specifically, it has been shown that a high Cu concentration resulted in the deficiency of other micronutrients, which adversely affected the yield of wheat plants [28]. Zinc (Zn) toxicity is related to Fe-deficiency-induced chlorosis through reductions in chlorophyll synthesis and chloroplast degradation, and interference with P, Mg and Mn uptake [29]. Vanadium (V) is a chemical analogue of phosphorus (P), and for this reason, it alters the P absorption capacity of plants [35]. An elevated nickel (Ni) level causes phytotoxicity, which influences nitrogen metabolism, ultimately expediting necrosis and senescence in plants [36,38]. In the same manner, chromium (Cr) toxicity has been associated to changes in the process of germination [42]. Therefore, the imbalance caused by heavy metals on the absorption and assimilation of other important nutrients directly affects plant growth and yield, and it is a serious problem for current agriculture. This heavy metal problem is increasing due to and along with industrialization and air/water pollution, so knowledge on which signaling mechanisms are affected by these heavy metal toxicities is essential to cope with this emerging problem.

## 3. Roles of MET in Metal Toxicity Tolerance

**Figure 1:**
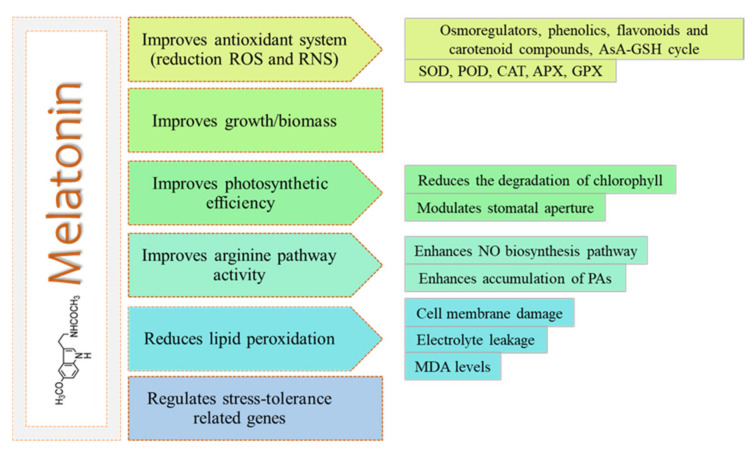
Common MET functions in metal toxicity tolerance. Reactive oxygen species (ROS), reactive nitrogen species (RNS), ascorbic acid (AsA), glutathione (GSH), catalase (CAT), superoxide dismutase (SOD), peroxidase (POD), ascorbate peroxide (APX), and glutathione reductase (GR), nitric oxide (NO), polyamines (PAs), malondialdehyde (MDA).

In general, the mechanisms that involve MET in the tolerance to metal toxicity are similar (Figure 1), although some MET-induced mechanisms are specific to a certain type of contaminant and plant species, which will be specifically described below. Thus, MET application improves photosynthesis efficiency, regulates metal transport and plant vegetative growth processes, decreases ROS and RNS levels and oxidative damage, and upregulates stress tolerance-related genes [43].

### 3.1. Regulation of Photosynthesis by the Application of Exogenous MET

Perhaps one of the most significant functions of MET in plants is the role it plays on increasing photosynthetic efficiency, as MET helps to improve the integrity of the photosynthetic pigments, such as chlorophyll, and increases the rate of photosynthetic electron transport chain (PET), and D1 protein synthesis. Thus, it has been shown that MET application enhanced photosynthetic efficiency under Pb, C, Cu, Zn, Al, Ni, or Cr toxicity (Table 1). In these studies, all the authors concluded that MET helped to improve plant growth under these metal toxicities due to an improvement in photosynthetic efficiency. 

### 3.2. MET Crosstalk with Other Plants Hormones

Several researchers have defined MET as a growth regulator or a phytohormone, as it can regulate plant vegetative growth processes such as rooting, flowering, leaf aging, photosynthetic yield, biomass yield, or the formation and maturation of seeds and fruits [13,14,15]. Thus, the phytohormone MET can crosstalk with other plant hormones (abscisic acid, gibberellin A14, zeatin, 24-epibrassinolide, and jasmonic acid (JA)) to regulate these physiological processes [72]. The combined transcriptomic and metabolomic analysis described by Hu et al. [73] revealed that MET could promote melon (*Cucumis melo*) root development by regulating linoleic acid metabolism. MET decreased the level of linoleic acid and the expression of four lipoxygenase (LOX)-related genes, thus decreasing the level of JA. Therefore, MET decreased ROS damage induced by Cu stress in melon plants by reducing LOX-related gene expression and JA levels, thus regulating the expression of other redox genes and increasing antioxidant enzyme activities that detoxified cellular ROS [73].

### 3.3. Regulation of Metal Transport by MET

**Figure 2:**
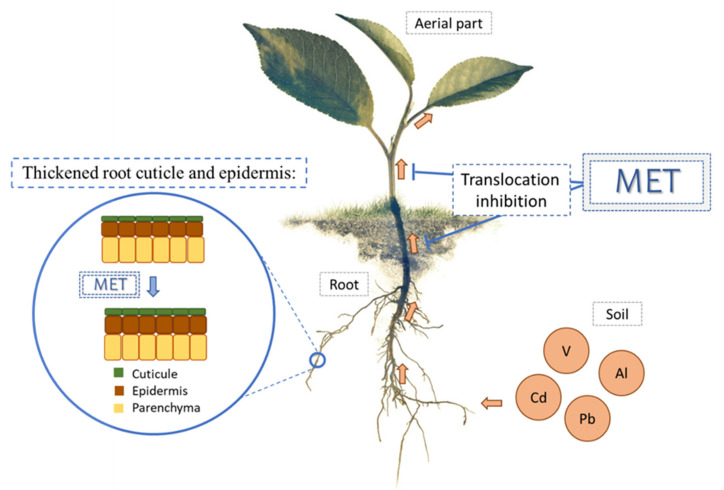
Regulation of metal transport by MET. Treatment of MET decreased Pb, Cd, V and Al transfer from root to aerial parts of the plant. In addition, exogenous MET was related to the thickened root cuticle and epidermis.

In several studies, MET has been shown to be involved in the regulation of metal transport in plants [59,62,67,74,75,76]. Namdjoyan et al. elucidated that MET application in safflower (*Carthamus tinctorius*) seedlings reduced Pb uptake and decreased Pb transfer from the root to the aerial parts of the plant [74] (Figure 2). Under the same stress, David et al. also demonstrated that the exogenous application of MET resulted in a thickened root cuticle and epidermis, which helped with the immobilization and localization of Pb to the root, and the decrease in the translocation of Pb to the leaves of *Amaranthus cruentus* [75] (Figure 2). In rapeseed (*Brassica napus*) seedlings, the analysis of Cd and Al in different subcellular compartments showed that MET restricted the mobilization of Al and Cd into vacuoles and the cell wall, and thus substantially decreased Al and Cd toxicity [62]. Similarly, in mallow (*Malva parviflora*) plants under Cd stress, low concentrations of MET led to a decrease in Cd translocation to the shoots [59]. In this way, Nawaz et al. (2018) showed that MET pretreatment of watermelon (*Citrullus lanatus*) seedlings increased the ability of the plants to accumulate V in the root tissues, thus reducing V transport from the root to stem and leaves [67] (Figure 2). However, in a Nazarian and Ghanati study [76], MET treatment of rice (*Oryza sativa*) plants under As stress was associated to As transport from roots to shoots, and therefore, a higher accumulation of As in shoots and suppression of the antioxidant system was observed. In another study, the effects of MET on the aquaporins (AQP) water channels were responsible for arsenic uptake and transport, which caused an intense increase in As concentrations in the aerial parts of rice plants. Both in the root and the aerial part, As adversely affected photosynthesis, growth, total sugar and protein production, and increased H_2_O_2_ content [76]. One of the essential characteristics of plants used in phytoremediation techniques is their ability to accumulate heavy metals into harvestable plant parts such as stems and leaves [74], with the aim of cleaning soils and/or waters of these metals. However, in most of the cases, MET improved the immobilization and the localization of metals to the root and decreased the translocation of metals to the leaves, which may help to not only clean soils and/waters from heavy metals, but also to commercialize the aerial parts of these plants for a safe human consumption, as they are clean of metals. In this sense, it is important to delve into the knowledge on the role of MET for the phytoremediation of contaminated soils and waters with edible plants/fruits.

Some more specific studies on metal stress tolerance in plants induced by MET have demonstrated that MET interacted with selenium (Se), Ca^2+^ or GSH to enhance metal toxicity tolerance. Li et al. [77] indicated that Se and MET supplements significantly increased Cd tolerance in tomato plants (*Solanum lycopersicum*) by optimizing plant growth parameters. Although exogenous selenocysteine could ameliorate Cd phytotoxicity, a basal level of endogenous MET was required for Se-conferred Cd tolerance, which may enhance the detoxification of Cd [77]. On the other hand, Goodarzi et al. [78] showed that the application of MET, GSH, and in particular, the combination of these two signaling molecules, could significantly reduce the dangerous effects of Zn-induced toxicity in safflower (*Carthamus tinctorius*) by reducing Zn accumulation in the shoots of safflower seedlings, and stimulating various antioxidant defense systems [78]. In addition, Siddiqui et al. [79] found that a combination of MET and Ca^2+^ was more efficient than their separate use to increase the tolerance of *Vicia faba* plants under metalloid As toxicity. Under As toxicity conditions, the application of MET and Ca^2+^ synergistically suppressed the apoptosis of stomata guard cells, DNA damage, and formation of ROS. Furthermore, it improved photosynthesis efficiency under these conditions. In addition, the expressions of ATP synthase, Ca^2+^-ATPase, Ca^2+^- DPKase, Hsp17.6 and Hsp40 were found at their maximum in plants treated with MET + Ca^2+^, resulting in a higher tolerance of plants to As stress. Ultimately, MET + Ca^2+^ treated plants conferred As toxicity tolerance shown as increased total soluble carbohydrates, cysteine, and proline (Pro) accumulation with increased Pro synthesizing enzyme (Δ1-pyrroline-5-carboxylate synthetase (P5CS), and decreased Pro degrading enzyme (Pro dehydrogenase) [79]. On the other hand, Zhang et al. [80] showed that an arbuscular mycorrhizal (AM) inoculation and MET application had a synergistic effect on host *Medicago truncatula* plant growth and Pb stress tolerance. In this synergy, AM inoculation may stimulate the accumulation of MET through the upregulation of ASMT (acetylserotonin methyltransferase, the enzyme that participates in the last step of the MET synthesis pathway) in roots. Moreover, the application of MET could improve mycorrhizal plant growth and Pb stress tolerance by improving AM symbiosis and stimulating an antioxidant response [80].

## 4. ROS and NO-Related MET Induced Stress Response in Plants

As described previously, plant responses to metal stress are complex. To reduce injury due to stress, plants have developed different pathways. The first stress response is the concomitant increase in ROS and RNS within the cells. ROS and RNS play an essential role as signaling molecules in the regulation of numerous biological processes such as growth, development, and abiotic and/or biotic stress responses in plants [81]. The temporal and spatial coordination between ROS and other signaling molecules is very well known as a primary mechanism of plant-related stress responses. Different studies have identified multiple core sets of genes and stress condition-dependent changes [81]. The levels of ROS and RNS are especially important in plants, as they are related to lipid peroxidation, electron leakage (EL), and resulting membrane damage, as well as damage to proteins and nucleic acids [82].

Transition metals, such as Cu, catalyze the formation of hydroxyl radicals from Fenton and Haber-Weiss reactions [83]. However, Cd and Hg do not seem to be able to intervene in these types of reactions, and although they are known to be potent inducers of oxidative stress [84,85], how this stress is induced is still unknown. Under heavy metal stress, nicotinamide adenine dinucleotide phosphate (NADPH)- oxidase is perhaps the main source of H_2_O_2_ and ROS that mainly accumulate in the apoplast after O_2_^•−^ generation [86,87]. In pea plants, exposure to Cd has been shown to generate a response that was characterized by an overproduction of ROS and a decrease in nitric oxide (NO) [86]. On the other hand, it has been shown that intracellular ROS production constantly increased in alfalfa seedlings exposed to Cd, with a lower increase in extracellular H_2_O_2_. Nevertheless, a small Hg treatment in epidermal cells of alfalfa roots caused an oxidative burst, as observed by a constant increase in extracellular H_2_O_2_ in roots, while intracellular ROS accumulated only temporarily [88]. Thus, Hg and Cd trigger different toxicity mechanisms [88]. In general, metals such as Cd, Cu, Fe, Zn, Hg, Mn, and Al have been shown to induce ROS production as a generalized response. Afterwards, ROS scavenging systems, including catalase (CAT), superoxide dismutase (SOD), peroxidase (POD), ascorbate peroxide (APX), and glutathione reductase (GR) often work together in the protection against excess ROS toxicity in plants [89].

Recently, it was shown that one of the molecules that was able to regulate the cellular concentration of ROS, was MET [17]. In this sense, it has been proposed that endogenous MET concentration is able to control ROS levels in two different ways: through its direct chemical interaction with ROS (i.e., ROS scavenger) which results in their detoxification [90,91,92]; or by the MET-mediated induction of the main antioxidant enzymes [93], such as SODs, APXs, and CATs, among others. As well, MET increases the accumulation of some representative non-enzymatic antioxidant compounds, such as GSH and ascorbic acid (AsA) [94,95,96,97,98,99,100,101], phenolic compounds [102], flavonoids via the nitric oxide-dependent (NO-dependent) pathway [103], and carotenoids [72,104], which help in ROS detoxification. Exogenous MET interacts with its main cellular receptor (CAND2/PMTR1), which could be MET-induced, leading to the activation of responses against stressors [105]. In addition, Arnao and Hernadez-Ruiz [92] showed that ROS can upregulate the MET biosynthesis genes and consequently, enhance the plant’s endogenous levels of MET, thereby directly helping in the antioxidant plant response [92].

### 4.1. ROS-Related MET Induced Stress Response in Plants

**Figure 3:**
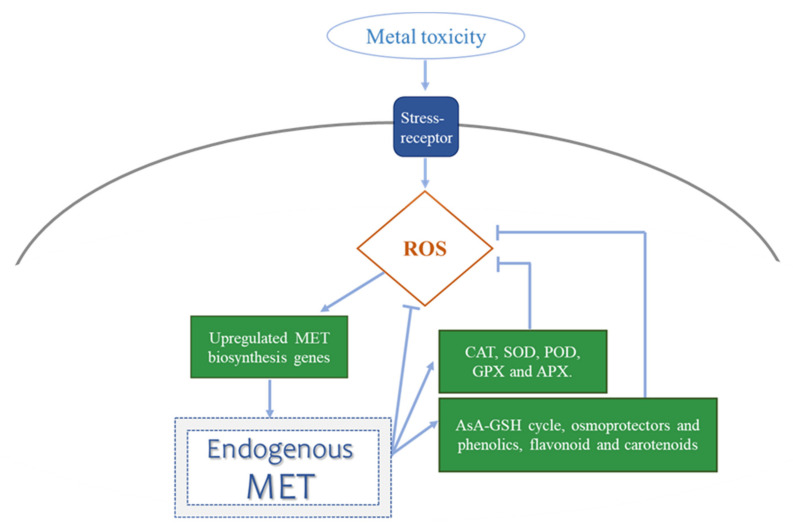
Interaction between melatonin (MET) and reactive oxygen species (ROS). ROS upregulate MET biosynthesis genes and enhance MET endogenous levels. MET can act as a ROS scavenger and control ROS levels through the melatonin-mediated induction of redox enzymes, such as CAT, SOD, POD, GPX and APX, as well as non-enzymatic antioxidant compounds, such as GSH and AsA (AsA-GSH cycle), osmoprotectants, and phenolic, flavonoid and carotenoid compounds.

Aside from its role as an antioxidant molecule, endogenous and exogenous MET are associated with a decrease in ROS levels and an increase in redox homeostasis due to the enhanced scavenging activity or the expression of some antioxidant enzymes, such as CAT, SOD, POD, GPX and, APX under most of the metal toxicity studies with metals including Pb, Cd, Cu, Zn, B, Al, V, Ni, As, or Cr (Table 2). The increased activity of antioxidant enzymes decreased EL, lipid peroxidation, malondialdehyde (MDA) content, and ROS content in the plants exposed to metal stress [59,64], which are usually increased and related to cell damage induced by stress (Figure 3).

The increased activity of antioxidant enzymes decreased EL, lipid peroxidation, malondialdehyde (MDA) content, and ROS content in the plants exposed to metal stress [59,64]. Moreover, MET supplementation has been shown to improve representative non-enzymatic antioxidant molecules, such as GSH and AsA (AsA-GSH cycle) under stress due to Pb, Cd, Cu, B, Ni, As, or Cr (Table 2) (Figure 3). Furthermore, a disturbance in the redox potential of tissues under metal toxicity results in the accumulation of various osmolytes and the activation of antioxidant compounds. Osmotic potential is regulated by osmolytes within plant tissues, e.g., in *Silene vulgaris*, Cd was shown to inhibit water transport and originated higher proline levels, thus avoiding Cd-induced lipid peroxidation [114]. The accumulation of proline in a plant is considered as a physiological adaptation under environmental stresses [115]. At the cellular level, variations in the concentrations of osmolyte can lead to a series of modifications in the active constituents (e.g., pectin, lipid, and protein) of the cell wall and the cytomembrane [116,117]. Additionally, the complexation of major osmolytes with intracellular metal ions can convert the chemical forms of the metal in cells, which are closely associated with the translocation and subcellular distribution of the metal [118,119,120,121]. In this sense, exogenous MET was shown to increase the concentration of important molecules associated with cell osmoregulation, such as some carbohydrates (trehalose) and amino acids (Pro), which are commonly accumulated to protect plants against metal stress such as Cd, Cu, B, Al, or As toxicity (Table 2). It has also been described that in mallow (*Malva parviflora*) plants under Cd toxicity, a MET treatment increased phenylalanine ammonia-lyase (PAL) activity, a key enzyme related to defense reactions and the main step in the phenylpropanoid synthesis pathway. These authors argued that an increase in shoot soluble carbohydrates could be related to an increased content of phenols in these plants under Cd toxicity [59]. This was also described for *Vicia faba* plants, where the exogenous application of MET induced the accumulation of total soluble carbohydrates, cysteine, and Pro, with the concomitant increase of the Pro-synthesis enzyme (Δ1-pyrroline-5-carboxylate synthetase, P5CS), and a decrease in the Pro-degrading enzyme (Pro dehydrogenase—PDH) [79].

Other non-enzymatic antioxidant molecules which have been shown to increase their concentration after an exogenous application of MET are phenolic compounds, flavonoids, and carotenoid compounds via the NO-dependent pathway [30,72,104,113,122]. The application of MET in wheat (*Triticum aestivum*) under high levels of B significantly reversed the adverse effects of B toxicity and alleviated the cellular oxidative damage through enhanced ROS scavenging, by the induction of some important antioxidant enzymes, the increase in AsA and GSH content, and the content of phenolic compounds [30]. Also, in pepper plants (*Capsicum annuum*) grown under B toxicity, an exogenous application of MET reversed the toxic effect of B by moderating B accumulation and increasing carbohydrate, carotenoid, and flavonoid contents in leaves and fruits, with the concomitant increase in photosynthetic activity and plant growth [113]. Similar results and conclusions have been obtained in spinach plants (*Spinacia oleracea*) exposed to high B concentrations [122].

Furthermore, some studies have shown that MET addition to the growth media improved arginine pathway activity, and consequently, the concentration of endogenous polyamines (PAs) increased under several types of abiotic stress, including metal toxicity [98,123,124,125]. In this sense, in cucumber (*Cucumis sativus*) plants grown under Cd toxicity, the addition of 2-hydroxymelatonin (2-OHMET) enhanced photosynthetic rate, intercellular CO_2_ concentration, stomatal conductance, and the activity of PA-biosynthesis enzymes (putrescine, spermidine and spermine), while at the same time reducing PA oxidase activity. 2-OHMET also reduced Cd toxicity through the upregulation in the expression of SOD, CAT, and APX and improved antioxidant scavenger activity to reduce H_2_O_2_, EL, and MDA in these plants [63].

Finally, some researchers have confirmed that MET significantly upregulated stress tolerance-related genes. Kobylińska and Posmyk [126] observed that a MET treatment on Pb-exposed *Nicotiana tabacum* line Bright Yellow 2 (BY-2) suspension cells increased the cells’ viability, and this beneficial effect was correlated with a drastic decrease in H_2_O_2_ concentration and lipid peroxidation but also with a change in the expression of the BI-1 protein (an accepted regulator of plant cell death) [126]. Additionally, a recent investigation conducted by Wang et al. [127] revealed that the foliar application of MET in tobacco (*Nicotiana tabacum*) leaves enhanced Cd tolerance by improving antioxidant defense activities, promoting cell wall or vacuolar sequestration of Cd and changing the expression of Cd-related genes (IRT1, Nramp1, HMA2, HMA4, and HMA3) [127]. In this way, Xu et al. firstly demonstrated that various candidate differentially-expressed genes encoding yellow stripe 1-like (YSL), heavy metal ATPases (HMA), and ATP-binding cassette (ABC) transporters were essential in the stress tolerance response, as they are involved in MET-mediated regulatory networks of Cd transportation and sequestration in radish (*Raphanus sativus*) roots. Xu et al. [128] also showed that the exogenous MET conferred Cd tolerance by the upregulation of the RsMT1 gene in radish plants [128]. As well, in MET-treated Chinese cabbage (*Brassica campestris* spp. *chinensis*) plants the concentration of Cd and the expression levels of related transport gene IRT1 were significantly reduced [61]. The transcriptome analysis by Cao et al. [65] demonstrated that MET broadly altered the expressions of various genes in cucumber (*Cucumis sativus*) under Cu stress. MET increased the levels of GSH and phytochelatin to chelate excess Cu, and improved cell wall trapping, thus keeping more Cu in the cell wall and in the vacuole, thereby reducing its cellular toxicity. MET inhibited ROS production and enhanced antioxidant systems at the transcriptional level and enzymatic activities [65]. Furthermore, at the transcriptomic and metabolomic levels, Hu et al. [73] showed that there were 70 significant differentially expressed genes (DEGs) (28 upregulated, 42 downregulated) and 318 significantly differentially expressed metabolites (DEMs) (168 upregulated, 150 downregulated) between the MET and the no-MET treatments in melon plants under Cu stress. Thus, these authors demonstrated that MET could promote melon root development by regulating the metabolism of linoleic acid. MET decreased the level of linoleic acid and the expression of four lipoxygenase (LOX)-related genes, thus reducing the JA level. MET decreased ROS damage by decreasing LOX-related gene expression and JA accumulation, enhancing antioxidant enzyme activities, and modulating the expression of other redox genes. Moreover, MET increased GSH, which diminished excess Cu^2+^. MET also regulated the expression of genes related to cell wall formation mechanisms, and AP2/ERF, BBR/BPC, GRAS, and HD-ZIP transcription factor families. Then, these processes were related to MET-alleviated copper toxicity and promoted melon root development [73]. Very recently, Li et al. [129], showed that in tea (*Camellia sinensis*) plants, a MET treatment alleviated As phytotoxicity through the increase in anthocyanins due to the exogenous MET upregulating the expression of anthocyanin biosynthetic genes such as CsCHS and CsANS, as anthocyanins have a potential function in ROS detoxification and metaloid chelation. Interestingly, the analysis of As content suggesting that MET improved As tolerance was dependent on the basal levels of anthocyanins in tea plants [129].

### 4.2. NO-Related MET-Induced Stress Response in Plants

Nitric oxide (NO) is another key signaling molecule in plant physiology. The signaling role of NO in plants has also been reported to regulate plant growth under control and stress conditions [130]. Treatment with exogenous NO prevents damage from stress, promotes disease tolerance, improves the nutritional quality of fruits, and delays fruit ripening [131]. NO plays an important role in the regulation of the cellular redox balance in plant cells through post-translational modifications (PTMs) and/or through its binding to the prosthetic heme group of a few antioxidant enzymes. These PTMs include S-nitrosylation, tyrosine nitration, and metal nitrosylation, with the first two being the most important at a physiological level [132]. Protein tyrosine nitration (NO_2_-Tyr) consists of the addition of a nitro (−NO_2_) group to one of the two equivalent ortho carbons of the aromatic ring of tyrosine residues [133]. S-nitrosylation is the covalent binding of NO to the thiol group of cysteines [134,135]. NO_2_-Tyr and S-nitrosylation can alter protein functions through a gain, no change, or loss of function, with the latter being the most common in plants [132,136]. Several studies have shown an interrelationship between S-nitrosylation and NO_2_-Tyr in the regulation of the activity of some antioxidant proteins, being an important mechanism for maintaining the antioxidant capacity of the AsA/GSH (ascorbic acid/glutathione) cycle under nitro-oxidative conditions [137,138]. Also, NO also regulates other important proteins related to other cellular processes. NO functions as a Ca^2+^-mobilizing messenger by promoting the rise in cytosolic Ca^2+^ concentrations. By increasing cytosolic Ca^2+^ concentration, NO regulates the activity of protein kinases and Ca^2+^-sensitive channels, which might be involved in the signaling cascade that causes the expression of defense-related genes (tolerance response to biotic and abiotic stresses), stomatal closure, or adventitious root formation, and germination. These processes involve cyclic adenosine diphosphate (cADP) ribose, cyclic guanosine monophosphate (cGMP), and protein kinases [139].

**Figure 4:**
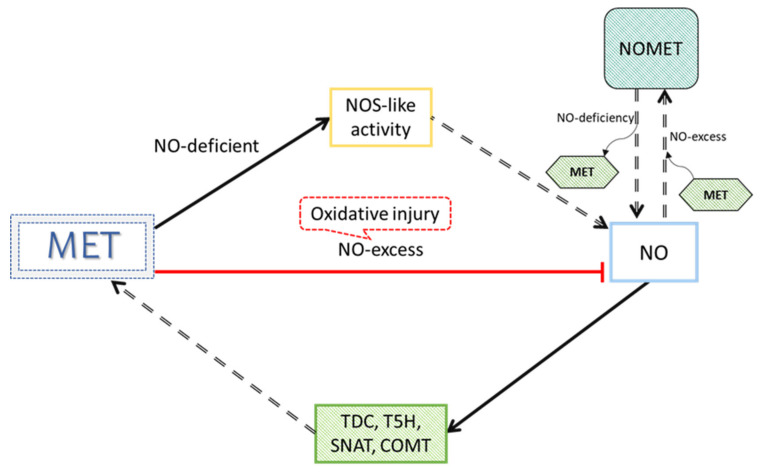
Interaction between melatonin (MET) and nitric oxide (NO). MET promotes the accumulation of NO by increasing the activity of NOS (nitric oxide synthase) by MET-mediated up-regulation of related genes. MET scavenges excess NO, as it produces oxidative injury (red arrow). In the presence of oxygen, MET can be easily converted to N-Nitrosomelatonin (NOMET) by NO nitrosation under different pH conditions, being NOMET an effective NO donor in cell cultures under the presence of serotonin and its derivatives. On the other hand, through a cyclic guanosine monophosphate (cGMP)-dependent pathway, NO induces the expression of TDC, T5H, SNAT and COMT genes that codify for the MET biosynthesis pathway enzymes to increase MET levels (these two process has not been described in plants grown under metal toxicity, although something similar was shown under other abiotic stresses). Abbreviations: Tryptophan decarboxylase (TDC), tryptamine5-hydroxylase (T5H), serotonin N-acetyltransferase (SNAT), and caffeic acid O-methyltransferase (COMT). Modified figure from our previous article [16].

Recent studies have shown evidence of MET enhancing the NO biosynthesis pathway through the regulation of endogenous NO content, nitrate reductase (NR) and NO synthase-related activities (via the arginine pathway), and the expression of their related genes [98,125,140]. As well, it has also been demonstrated that NO can upregulate MET, through the direct regulation of this molecule of MET-related biosynthetic enzymes [94]. Also, NO regulates MET accumulation through the formation of N-Nitrosomelatonin (NOMET) [141]. In the presence of oxygen, MET can be efficiently converted to NOMET by NO nitrosylation under different pH conditions. Nevertheless, under the presence of serotonin and its derivatives, NOMET is an effective NO donor in cell cultures [142,143]. MET is transported in the form of a metabolic signal NOMET from the roots, across the hypocotyl, until reaching the cotyledon cells in less than 48 h after radicle emergence, leading to a reduction in both oxidative and nitrosative stress in sunflower seedlings under salt stress. That is, NO plays a role as a positive modulator of MET accumulation in seedling cotyledons in a long-distance signaling response [141]. More studies are needed to understand the interconnection between MET and NO. Nevertheless, a considerable number of studies have demonstrated that MET increases NO levels under abiotic stress [94,98,125,140,141,144,145,146,147] (Figure 4).

The MET and NO coordination enhances many metal toxicity-related tolerance in plants, such as Pb toxicity in maize [106], Cd toxicity in *Catharanthus roseus* [111,112,148], wheat (*Triticum aestivum*) [50] and Chinese cabbage (*Brassica campestris* spp. *chinensis*) [61], Zn toxicity in rice plants [149] and Al toxicity in *Arabidopsis thaliana* [150] plants. Nabaei and Amooaghaie [148] confirmed that co-treatment of MET and NO, improved Cd tolerance and phytoremediation efficiency *in Catharanthus roseus* plants. The pre-assumption of the additive effect of MET and NO or the hypothesis that these signal molecules have synergistic relationships for enhancing tolerance and accumulation of Cd in *C. roseus* plants [148] have also been shown. Studies in *C. roseus* showed that MET and sodium nitroprusside (SNP as a NO donor) significantly improved seedling growth by increasing the concentration of photosynthetic pigments, endogenous NO concentration in roots, Pro concentration, and the activities of antioxidant enzymes (SOD, POD, APX, and CAT). All of these were induced to lower H_2_O_2_ and lipid peroxidation levels in roots of *C. roseus* plants under Cd stress. On the other hand, it seemed that NO acted downstream of MET in modulating seed germination [112] and antioxidant responses in roots [111] of *C. roseus* plants under Cd toxicity. In MET-treated Chinese cabbage (*Brassica campestris* spp. *chinensis* plants, Wang et al. showed a significantly reduced concentration of Cd and expression levels of related transport genes IRT1 (regulators of Cd absorption). Under Cd toxicity, NO increases the expression of IRT1, thus further increasing the absorption of Cd and intensifying the stress of Cd in plants, while an exogenous treatment with MET under Cd toxicity inhibits the synthesis of NO, and therefore, MET reduces the Cd content in the plant and Cd toxicity as well [61]. Huang et al. revealed that MET application substantially increased dry biomass accumulation, root growth, mineral absorption, and antioxidant responses of rice under various levels of ZnO nanoparticle application. The NO induced in rice plant plays an important role in producing resistance against ZnO nanoparticle toxicity by regulating MET metabolism and antioxidant enzyme activities. Their comparative transcriptome analysis also identified key genes which were responsible for MET and NO-induced modulations in plant growth under ZnO nanoparticle toxicity [149]. Additionally, pharmacological and genetic evidence in *Arabidopsis thaliana* also suggested that exogenous and endogenous MET concentrations were involved in the alleviation of Al toxicity-induced root growth inhibition, through the interference of the NO signaling pathway [150]. Finally, Zhang et al. demonstrated that the expression of serotonin N-acetyltransferase (SNAT), encoding a key enzyme involved in MET synthesis, was downregulated by Al, which coincided with decreased MET accumulation in *Arabidopsis thaliana*. That is, SNAT-mediated MET synthesis played a critical role in Al toxicity resistance [150].

## 5. Conclusions and Future Perspectives

In plants melatonin (MET) is associated to numerous functions, among which we find the regulation of physiological processes such as flowering or rooting, and its involvement in tolerance to abiotic and biotic stress conditions. MET alleviates metal stress or metal toxicity directly through the scavenging of ROS and RNS, and indirectly through the enhancement of antioxidant activities and photosynthetic capacity, the regulation of plant growth regulators, the increase of osmotic metabolites, the regulation of metal transport, and the downregulation or upregulation of stress-related genes in plants. However, relatively few studies have been focused on the genes and core pathways that are specifically regulated by MET. In addition, several researchers have revealed that MET is involved in the signaling pathway that is directly mediated by NO, although their relationship is still confusing. More research works are needed to comprehend the relation between endogenous MET and NO, as most studies have only focused on exogenous MET. In this sense, no studies have been found that showed the implication of the relationship between MET and abscisic acid (ABA), or ethylene in plants, on the tolerance to metal toxicity. However, different studies have demonstrated that ABA-dependent pathways may have contributed to MET-induced cold and salinity tolerance [151,152] and that ethylene production was suppressed by MET through the downregulation of the ethylene biosynthesis-related genes under waterlogging stress in plants [124]. On the other hand, apart from the toxicity produced by the metals mentioned in this review, it has been observed that MET can also increase the tolerance in plants subjected to stress due to pollutants such as lanthanum [153], selenium [154], fluoride ions [155,156], and bisphenol A [BPA; 2,2-bis(4-hydroxybenzene)] [157], which will need further investigation due to the importance of these toxic molecules for human health. Therefore, the knowledge of the different signaling mechanisms that involve MET in heavy metal detoxification could lead to new ways to improve our future agriculture, to create products with high nutritional value, to obtain soils and waters that are less contaminated, and lastly, to increase human health.

## Figures and Tables

**Table 1:** Selected studies on the roles played by melatonin on the photosynthesis efficiency response of plants to metal toxicity.

Metal Toxicity	Plant Species	References
Pb	bermudagrass (*Cynodon dactylon*)	[44]
	*Eruca vesicaria*	[45]
Cd	alfalfa (*Medicago sativa*)	[46]
	tomato (*Solanum lycopersicum*)	[47,48,49]
	wheat (*Triticum aestivum*)	[50,51]
	*Cyphomandra betacea*	[52]
	*Malachium aquaticum*	[53]
	*Galinsoga parviflora*	[53]
	*Perilla frutescens*	[54]
	rice (*Oryza sativa*)	[55,56]
	*Ulva* (green macroalga)	[57,58]
	mallow (*Malva parviflora*)	[59]
	*Spinacia oleracea*	[60]
	Chinese cabbage (*Brassica campestris* spp. *chinensis*)	[61]
	rapeseed (*Brassica napus*)	[62]
	cucumber (*Cucumis sativus*)	[63]
	strawberries (*Fragaria* × *ananassa*)	[64]
Cu	cucumber (*Cucumis sativus*)	[65]
Zn	wheat (*Triticum aestivum*)	[66]
Al	rapeseed (*Brassica napus*)	[62]
V	watermelon (*Citrullus lanatus*)	[67]
Ni	tomato (*S. lycopersicum*)	[68]
Cr	wheat (*Triticum aestivum*)	[69]
	canola (*Brassica napus*)	[70,71]

**Table 2:** Selected studies on the roles played by MET treatment related with a decrease in ROS levels and an increase in redox homeostasis, due to the enhanced scavenging activity or the expression of some antioxidant enzymes, enhanced non-enzymatic antioxidant molecules, such as GSH and AsA (AsA-GSH cycle) and cell osmoegulation.

ROS Regulation	Metal Toxicity	Plant Species	References
Antioxidant enzymes	Pb	bermudagrass (*Cynodon dactylon*)	[44]
		maize (*Z. mays*)	[106]
		*Ulva* (green macroalga)	[58]
	Cd	mallow (*Malva parviflora*, Malvaceae)	[59]
		*Spinacia oleracea*	[60]
		strawberries (*Fragaria* × *ananassa*)	[64]
		alfalfa (*Medicago sativa*)	[46]
		tomato (*Solanum lycopersicum*)	[47,48,49]
		wheat (*Triticum aestivum*)	[50,51]
		*Cyphomandra betacea*	[52]
		*Malachium aquaticum*	[53]
		*Galinsoga parviflora*	[53]
		*Perilla frutescens*	[54]
		rice (*Oryza sativa*)	[55,56]
		*Ulva* (green macroalga)	[57,58]
		rapeseed (*Brassica napus*)	[62]
		cucumber (*Cucumis sativus*)	[63]
	Cu	cucumber (*Cucumis sativus*)	[65]
		melon (*Cucumis melo*)	[73]
	Zn	*Ulva* (green macroalga)	[58]
		wheat (*Triticum aestivum*)	[60]
		safflower (*Carthamus tinctorius*)	[78]
	Al	soybean (*Glycine max*)	[107]
		Wheat (*Triticum aestivum*)	[108]
		rapeseed (*Brassica napus*)	[62]
	V	watermelon (*Citrullus lanatus*)	[67]
	Ni	tomato (*S. lycopersicum*)	[68]
	Cr	wheat (*Triticum aestivum*)	[69]
		canola (*Brassica napus*)	[71]
	B	wheat (*Triticum aestivum*)	[30]
	As	rosemary (*Rosmarinus officinalis*)	[109]
		rice (*Oryza sativa*)	[41,76]
AsA-GSH cycle	Pb	bermudagrass (*Cynodon dactylon*)	[44]
		maize (*Z. mays*)	[106]
		*Ulva* (green macroalga)	[58]
	Cd	mallow (*Malva parviflora*, Malvaceae)	[59]
		cucumber (*Cucumis sativus*)	[110]
		strawberries (*Fragaria* × *ananassa*)	[64]
	Cu	cucumber (*Cucumis sativus*)	[65]
	Ni	tomato (*S. lycopersicum*)	[68]
	Cr	wheat (*Triticum aestivum*)	[69]
		canola (*Brassica napus*)	[71]
	B	wheat (*Triticum aestivum*)	[30]
	As	rosemary (*Rosmarinus officinalis* L.)	[109]
		rice (*Oryza sativa*)	[41,76]
Osmoregulation by carbohydrates (trehalose) and amino acids (proline) regulation	Cd	*Catharanthus roseus*	[111,112]
		*Brassica napus*	[62]
		mallow (*Malva parviflora*)	[59]
	Cu	melon (*Cucumis melo*)	[73]
	Al	*Brassica napus*	[62]
	B	pepper (*Capsicum annuum*)	[113]
	As	*Vicia faba*	[79]
		rosemary (*Rosmarinus officinalis*)	[109]
